# Rapid and Sensitive PCR-Dipstick DNA Chromatography for Multiplex Analysis of the Oral Microbiota

**DOI:** 10.1155/2014/180323

**Published:** 2014-11-17

**Authors:** Lingyang Tian, Takuichi Sato, Kousuke Niwa, Mitsuo Kawase, Anne C. R. Tanner, Nobuhiro Takahashi

**Affiliations:** ^1^Division of Oral Ecology and Biochemistry, Tohoku University Graduate School of Dentistry, Sendai 980-8575, Japan; ^2^Department of Prosthodontics, West China Hospital of Stomatology, Sichuan University, No. 14, Section 3, South Renmin Road, Chengdu 610041, China; ^3^Tohoku University Graduate School of Biomedical Engineering, Sendai 980-8579, Japan; ^4^Future Technology Management Center, Corporate R&D, NGK Insulators, Mizuho, Nagoya 467-8530, Japan; ^5^Department of Microbiology, The Forsyth Institute, Cambridge, MA 02142, USA

## Abstract

A complex of species has been associated with dental caries under the ecological hypothesis. This study aimed to develop a rapid, sensitive PCR-dipstick DNA chromatography assay that could be read by eye for multiplex and semiquantitative analysis of plaque bacteria. Parallel oligonucleotides were immobilized on a dipstick strip for multiplex analysis of target DNA sequences of the caries-associated bacteria, *Streptococcus mutans*, *Streptococcus sobrinus*, *Scardovia wiggsiae*, *Actinomyces* species, and *Veillonella parvula*. Streptavidin-coated blue-colored latex microspheres were to generate signal. Target DNA amplicons with an oligonucleotide-tagged terminus and a biotinylated terminus were coupled with latex beads through a streptavidin-biotin interaction and then hybridized with complementary oligonucleotides on the strip. The accumulation of captured latex beads on the test and control lines produced blue bands, enabling visual detection with the naked eye. The PCR-dipstick DNA chromatography detected quantities as low as 100 pg of DNA amplicons and demonstrated 10- to 1000-fold higher sensitivity than PCR-agarose gel electrophoresis, depending on the target bacterial species. Semiquantification of bacteria was performed by obtaining a series of chromatograms using serial 10-fold dilution of PCR-amplified DNA extracted from dental plaque samples. The assay time was less than 3 h. The semiquantification procedure revealed the relative amounts of each test species in dental plaque samples, indicating that this disposable device has great potential in analysis of microbial composition in the oral cavity and intestinal tract, as well as in point-of-care diagnosis of microbiota-associated diseases.

## 1. Introduction

With increasing appreciation of the role of bacterial communities in the etiology of dental caries [1], information about the qualitative and quantitative composition and changes in oral biofilms are needed to understand the infection associated with disease initiation and progression [2–4]. The development of a rapid, simple, accurate point-of-care techniques for oral microbiota profiling, particularly the detection and quantification of oral pathogens, has thus become a matter of urgency. The advent of polymerase chain reaction (PCR) initiated a revolution in clinical microbiology from traditional approaches, such as microscopy, culture, antigen detection, and immunoserology, to molecular techniques predominated by nucleic acid amplification- (NAA-) based methods [5, 6]. PCR amplification provides good specificity, sensitivity, reproducibility of results, and a basis for the development of assays that exploit differences in DNA sequences. Substantial efforts have been made to optimize PCR methods [7], and different protocols including standardized direct PCR, multiplex PCR, and the more sensitive nested PCR have been developed and applied to oral microbiology [8–14].

Simplicity is another advantage of PCR. NAA-based techniques avoid the need for in vitro cultivation and shorten the time to detect species from days to several hours. However, conventional post-PCR analysis using gel electrophoresis with ethidium bromide staining is hazardous, labor-intensive, and time-consuming and may not be sufficiently specific and sensitive. Advanced probe or capture hybridization methods developed in partial response to these drawbacks still require multiple procedures including heat denaturation, hybridization and washing, and expensive instrumentation [15]. DNA biosensors provide an alternative approach to simplifying post-PCR analysis with greatly enhanced detectability, specificity, and reproducibility. The general principle of DNA biosensors involves the immobilization of a DNA probe on the surface of the sensor, with hybridization and detection by electrochemical [16], optical [17], or gravimetric means [18]. PCR can be carried out with primers with bulky substituents bound to their 5′ or 3′ ends, which is useful for the design of DNA biosensors. Specific oligonucleotide sequences or immunologic substances (e.g., hapten-antibody and hapten-protein) that offer extremely strong affinity to capture DNA amplicons have been investigated as DNA probe candidates. More recently, progress on DNA biosensors has enabled visual detection of DNA by employing gold nanoparticles [19–22] and colored polystyrene beads [23] as signal generators. Quantitative data can be recorded by intensity readout or by additional coupling with an enzyme [20], fluorescent dye [24, 25], or radioactive conjugates [26]. Further developments of DNA biosensors have exploited more powerful strategies including quantitative real-time PCR [27–31], DNA microarrays [32], and surface plasmon resonance [33, 34]. These techniques have emerged as novel quantitative methods in microbiology but are expensive and require trained laboratory technicians.

A growing number of DNA biosensors have been designed for point-of-care diagnosis, in the form of lateral flow (dipstick) nucleic acid test strips [19–24, 26, 36]. Paper-based microfluidic bioassay appears to meet all the key standards of a point-of-care diagnostic device [37], that is, a sensitive and accurate assay that requires no complex equipment, reagents, or power sources and, preferably, a method that is rapid, small, disposable, and easy to use and transport.

This study aimed to develop a combination of PCR and dipstick-type DNA biosensors into one system as a rapid, sensitive, and visible multiplex analysis device to profile the oral microbiota, targeting the caries-associated bacteria;* Streptococcus mutans*,* Streptococcus sobrinus*,* Scardovia wiggsiae*,* Actinomyces* species, and* Veillonella parvula*. These bacteria have been known as crucial bacteria in the oral cavity as follows. Mutans streptococci, in particular,* S. mutans* and* S. sobrinus*, have been thought to be associated with dental caries, since they have frequently been isolated from dental plaque biofilm where they produce large amounts of acids and extracellular polysaccharides, which promote dental caries. It has been reported that* S. wiggsiae* has a strong association with early childhood caries in the USA [38].* Actinomyces* species are predominantly detected from dental plaque biofilm, periodotitis lesions, and root surface caries [39].* Veillonella* species, such as* Veillonella parvula*, are one of the indigenous bacteria of the oral cavity (dental plaque biofilm and tongue coating), and the species obtain their energy by fermenting organic acids, for example, lactate [40]. This metabolism has the potential to remove a potent, dental-caries producing acid. Most of the current DNA biosensors utilize streptavidin-biotin interaction as the DNA capture mechanism, allowing only single detection of a target dsDNA in one test. To simultaneously test for several bacterial species, different oligonucleotide strands were immobilized in parallel on one strip. In addition, streptavidin-coated colored latex microspheres were used as reporters to give chromatic signals. Semiquantitative information based on visual intensity was attempted by a standardized 10-fold serial-dilution without any specialized reagents or instrumentation.

## 2. Materials and Methods

### 2.1. Bacterial Strains

Five bacterial strains,* Streptococcus mutans* NCTC 10449,* Streptococcus sobrinus* 6715,* Scardovia wiggsiae* F0424,* Actinomyces oris* WVU627, and* Veillonella parvula* ATCC 10790, were cultured on CDC anaerobic 5% sheep blood agar (BD, Franklin Lakes, NJ) plates and were incubated at 37°C for 3 days in an anaerobic glove box (Model AZ-Hard, Hirasawa, Tokyo, Japan) under an atmosphere of 80% N_2_, 10% H_2_, and 10% CO_2_.

### 2.2. Subjects and Sample Collection

After obtaining informed consent, supragingival plaque (1.5 mg) from caries-free enamel surfaces was sampled from 16 healthy subjects (mean age, 31.8 ± 8.9 years; range, 23–54 years). The dentition (dental caries) status was recorded using the DMFT index (decayed, missing, and filled teeth); and third molars were excluded. No subjects had taken antibiotics for 3 months prior to sampling. Plaque samples were collected using sterile toothpicks and stored in 1.5 mL sampling tubes at −20°C before analysis.

### 2.3. DNA Extraction

Genomic DNA was extracted from dental plaque or 3-day-old bacterial cultures using the InstaGene Matrix Kit (Bio-Rad Laboratories, Richmond, CA) according to the manufacturer's instructions.

### 2.4. Polymerase Chain Reaction

Direct PCR was performed using the tagged species-specific primers listed in Table 1. Each PCR mixture comprised 21 *μ*L of either plaque sample DNA or bacterial genomic DNA, 25 *μ*L of Taq DNA polymerase (HotStar* Taq* PLUS Master Mix; Qiagen GmbH, Hilden, Germany), and 4 *μ*L of primer mixture. PCR amplification was conducted in a PCR Thermal Cycler MP (TaKaRa Biomedicals, Ohtsu, Shiga, Japan) or an iCycler (Bio-Rad Laboratories) programmed for 5 min at 95°C initial heat activation and 30 cycles of 1 min at 95°C for denaturation, 1 min at appropriate temperatures (in Table 1) for annealing and 1.5 min at 72°C for extension, followed by 10 min at 72°C for final extension.

### 2.5. Dipstick DNA Chromatography

**Figure 1:**
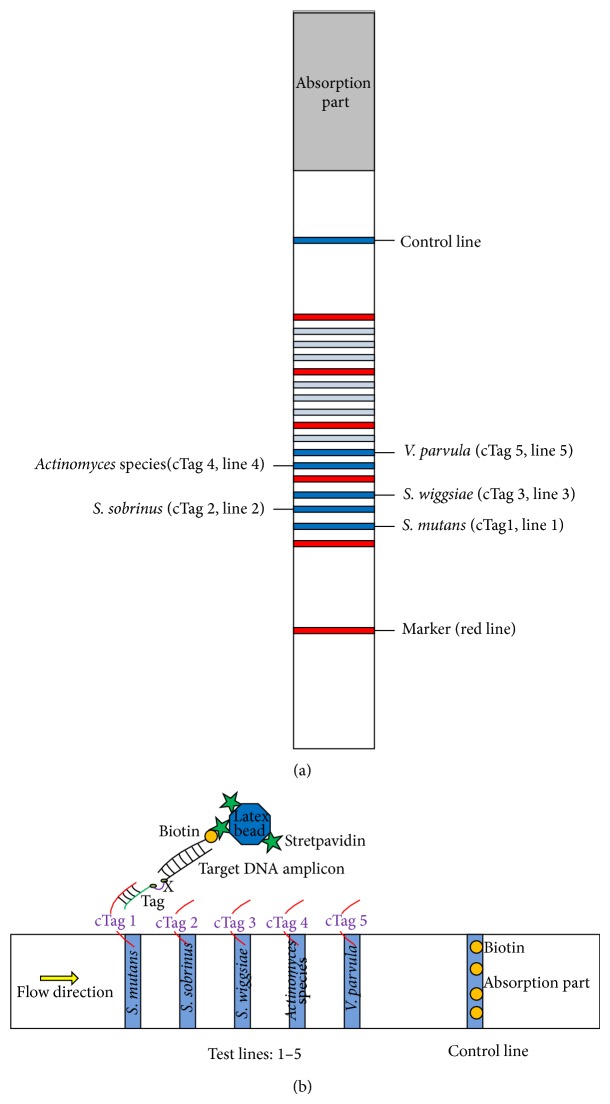
(a), (b) Schematic diagram of dipstick DNA chromatography. Tag = oligonucleotide strand; cTag = complementary oligonucleotide strand.

Dipstick DNA strips and reagents were obtained commercially (TBA Co., Sendai, Japan). As shown in Table 1, for each pair of primers, the 5′ terminus (of forward primer) was tagged with five different oligonucleotides and the 5′ terminus (of reverse primer) was biotinylated. Because the Spacer C3 [three-carbon spacer, (CH_2_)_3_, using Phosphoramide C3 spacer (Glen Research, Sterling, VA)] (representing “X” in Figure 1(b) and Table 1) is inserted between Tags 1 (to 5) and a respective forward primer, the DNA synthesis by* Taq* DNA polymerase terminates at the insertion site [35]. Blue-colored latex particles coated with streptavidin were linked to biotinylated terminal amplicons through a streptavidin-biotin interaction. Dipstick strips (2.5 mm × 45 mm) were manufactured by immobilizing five complementary oligonucleotides to specifically recognize the PCR amplicons through hybridization with 5′ terminus tags. On the strip, 12 test lines in total were available and the first 5 in order were selected in the present study. A biotin-immobilized flow control line was set up at the end of the strip (Figure 1). One PCR-amplified product was diluted into a total volume of 30 *μ*L and mixed with 10 *μ*L of eluent (containing detergents, blocking agents, PBS, and salts solution) and 2 *μ*L of streptavidin-coated blue latex suspension (both of them were supplied by TBA Co.). A DNA strip was then inserted into the mixture. A visible blue test line (0.5 mm width; 1.2 mm apart from each other) would appear where the complementary oligonucleotide was immobilized indicating the presence of target DNA sequences.

### 2.6. Sensitivity of Dipstick DNA Chromatography

To validate the sensitivity of dipstick DNA chromatography, aliquots containing 10 ng, 1 ng, 100 pg, 10 pg, or 1 pg of DNA amplified by PCR from five bacterial strains were prepared and assayed in parallel. The detection limit of dipstick DNA chromatography for each bacterial strain was determined by the visual inspection.

### 2.7. Specificity and Sensitivity of PCR-Dipstick DNA Chromatography

The specificity of PCR-dipstick DNA chromatography was confirmed under the following conditions: (1) oligonucleotide-free primers; (2) biotin-free primers; and (3) no target DNA template. Cross reaction of the primers with other bacteria was previously examined by in vitro experiments using representative oral bacteria [10, 38, 41, 42]. In addition, in order to confirm the specificity of primers in silico, BLAST searches were performed in this study through the website of the National Center for Biotechnology Information. We found that the primers of* S. sobrinus* cross-reacted with* Streptococcus downei*, and those of* Actinomyces* species (*Actinomyces oris*,* Actinomyces naeslundii*, and* Actinomyces odontolyticus*) cross-reacted with* Micromonospora peucetia*; however,* S. downei* and* M. peucetia* have not been reported to be isolated from the human oral cavity.

In order to assess the assay sensitivity, serial 10-fold dilutions of purified DNA from each bacterial strain (range: 10 ng to 1 fg) were amplified by PCR and divided into 2 equal parts. One part was used for dipstick DNA chromatography and the detection limit was determined. The other part was assayed with 1% agarose gel electrophoresis (High Strength Analytical Grade Agarose, Bio-Rad Laboratories), as follows. PCR products (10 *μ*L each) were separated on 1% agarose gels in Tris-borate EDTA buffer (100 mM Tris, 90 mM borate, 1 mM EDTA, pH 8.4), stained with ethidium bromide, photographed under UV light. A 100-bp DNA Ladder (Invitrogen Corp., Carlsbad, CA) was used as a molecular size marker.

### 2.8. Semiquantification Assay Procedure for Plaque Samples

Semiquantitative analysis of clinical samples requires rigorous optimization and standardization. DNA was extracted from dental plaque samples and amplified by PCR for each test species (Table 2). PCR-amplified DNA was 10-fold diluted from the original concentration to a 10^4^ multiple, and a 1 *μ*L aliquot at the same dilution factor for each species was mixed and detected by dipstick DNA chromatography. The detection limit of each bacterial species in dental plaque was obtained from a serial chromatogram, and the relative concentration of each bacterial species was estimated based on the dilution fold for the detection limit, with correction using the relative sensitivity of PCR-dipstick DNA chromatography, as described above.

## 3. Results and Discussion

### 3.1. Validation Principle of Dipstick DNA Chromatography

**Figure 2:**
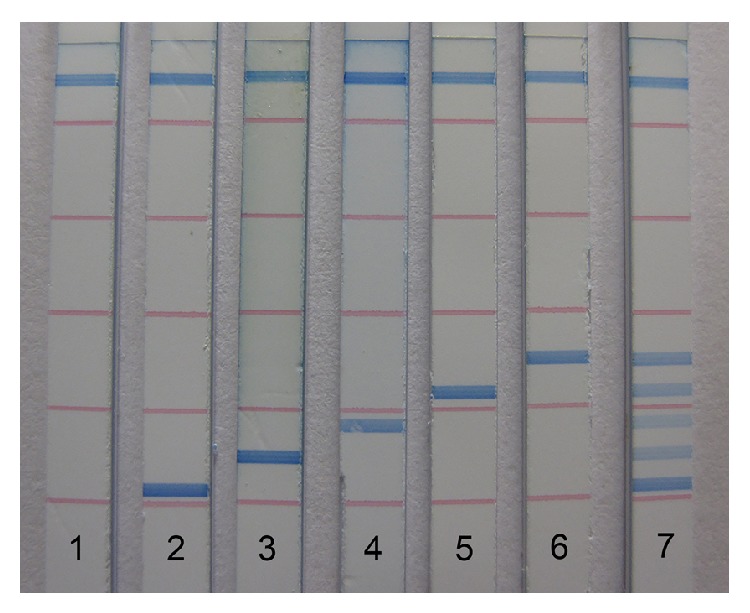
Dipstick-DNA chromatography. 1: negative control; 2–6: single detection of* Streptococcus mutans*,* Streptococcus sobrinus*,* Scardovia wiggsiae*,* Actinomyces oris*, and* Veillonella parvula*, respectively; 7: multiplex detection.

In the reaction mixture, the biotin-labeled PCR amplicon immediately attached to the streptavidin-coated blue latex particles due to the strong biotin-streptavidin affinity to form a hybrid. When the tip of the dipstick strip was inserted into the mixture, PCR amplicon-blue latex hybrids moved up through the strip by capillary action and were captured by tag-complement oligonucleotide fixed as test line on the strip, resulting in accumulation of blue latex microspheres to induce a visible blue test line. The flow control line on the other tip of strip was set up to confirm the proper function of the assay and also to capture excess latex particles, indicating that the amount of latex particles was capable of catching all the DNA amplicons. Different DNA amplicons could be detected either alone within 5 min, or simultaneously in one assay after 5–10 min with no cross reactions (Figure 2), thus confirming that this method is applicable for multiplex analysis.

The development of dipstick biosensors has entered into a more advanced stage which is focusing on enhancing the multiplexing capabilities. Biosensors with two or more test lines were subsequently proposed to simultaneously detect several alleles for genotyping of a biallelic polymorphism [43–45]. These biosensors involved either primer extension (PEXT) or oligonucleotide ligation reactions (OLR) which usually required an additional step in the assays. A method that detects the dsDNA could maintain the simplicity of a dipstick DNA biosensor. Such DNA biosensors have been reported by utilizing oligonucleotides to bind PCR amplicons with reporters and streptavidin-biotin as capture mechanism, which allow only one target dsDNA amplicon to be detected [19]. In contrast, in the present study, five different oligonucleotides complementary to corresponding tags were mounted on one strip, thus enabling multiplex detection of dsDNA sequences in one assay.

### 3.2. Sensitivity of Dipstick DNA Chromatography

**Figure 3:**
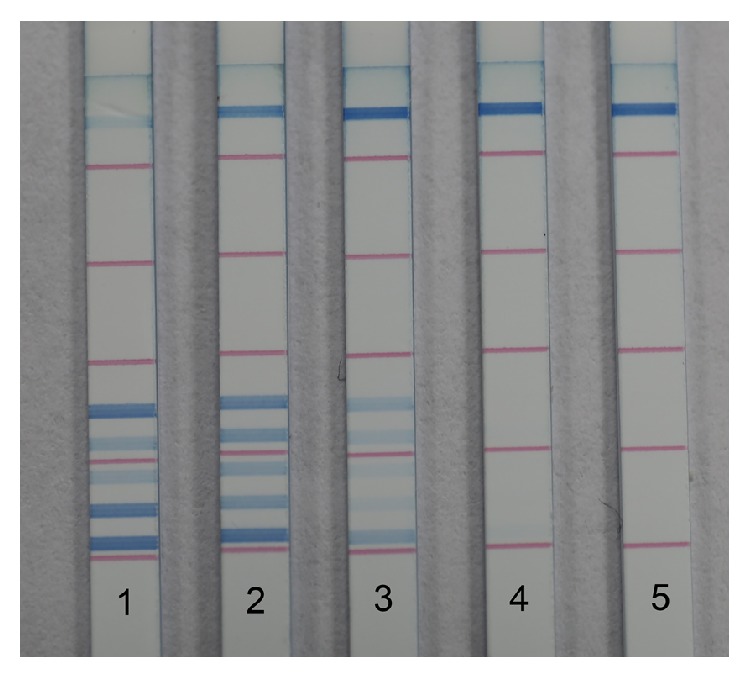
Detection limit of dipstick DNA chromatography. Amount of each target DNA amplicon detected on strips 1–5: 10 ng, 1 ng, 100 pg, 10 pg, and 1 pg.

The detection limit of dipstick DNA chromatography was evaluated using purified DNA amplified from each bacterial strain so as to exclude the influence of the PCR procedure on sensitivity (Figure 3). For all five species, PCR product could be detected at levels as low as 100 pg. Intensity was observed to be proportional to DNA concentration. When the amount of DNA product was tested in the useful analytical range below the saturation concentration, intensity provided quantitative information on target DNA [19], which laid the foundation for the following semiquantification analysis.

### 3.3. Specificity and Sensitivity of PCR-Dipstick DNA Chromatography

**Figure 4:**
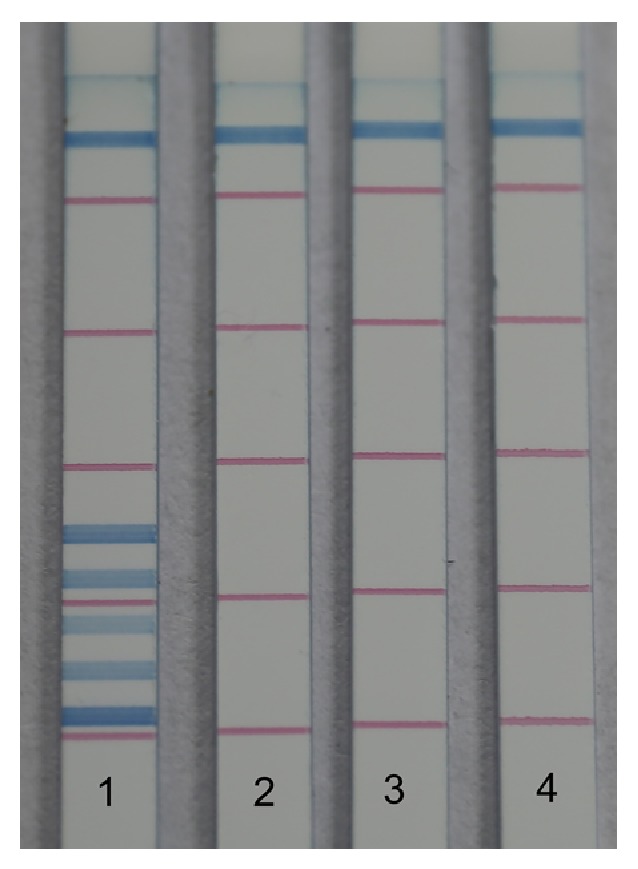
Specificity of PCR-dipstick DNA chromatography. 1: 5 target amplicons tagged by oligonucleotides at 5′ ends and biotin at 3′ ends. 2: target amplicons tagged by oligonucleotides only. 3: target amplicons tagged by biotin only. 4: PCR performed with no target DNA templates.

In this study, five test lines generated by target DNA hybrids appeared where expected. When five target DNA amplicons were detected in one assay, no smear or false positive lines were observed on the strip. The results of specificity assessment are presented in Figure 4. Analysis performed with oligonucleotide-free or biotin-free primers or in the absence of target DNA templates gave no positive signals. Under these three circumstances, the streptavidin-coated blue latex particles could not link to the oligonucleotide detectors to generate blue signal in the test zone and were finally captured in the control line. The specificity of dipstick DNA chromatography using the same biosensors was also confirmed by another project in which 176 bacterial strains were detected [46]. In addition, no cross reactions with other bacteria were observed in silico (in this study) or in vitro [10, 38, 41, 42], thus supporting the high specificity of this method.

A comparison of the detection sensitivity obtained using the PCR-dipstick DNA chromatography and PCR-agarose gel electrophoresis is shown in Table 3. Although there were differences among the five bacterial species, PCR-dipstick DNA chromatography showed at least 10-fold higher sensitivity than agarose gel electrophoresis, demonstrating the advantages of PCR-dipstick DNA chromatography.

### 3.4. Semiquantification of Dental Plaque Samples

The results from PCR-dipstick DNA chromatography using purified bacterial DNA showed that the sensitivity of this method differed between bacterial species (Table 3). As dipstick DNA chromatography exhibited the same detection limit for all five species (Figure 3), there were differences in detection sensitivity that could only be derived from PCR amplification efficiency. The relative PCR amplification efficiencies (**E**_**r**_) of* S. mutans*,* S. sobrinus*,* S. wiggsiae*,* A. oris*, and* V. parvula* were estimated to be 1, 10^−4^, 10^−3^, 10^−3^, and 10^−2^, respectively, from Table 3. Thus, relative concentrations of bacteria (**C**_**r**_) could be calculated by multiplying dilution fold for detection limit (**D**) and dividing by relative PCR efficiency (**E**_**r**_), using the following equation:

(1) $$\begin{array}{c} C_{r}=\frac{D}{E_{r}} \end{array}$$

**C**_**r**_ is relative concentration of a given bacteria in dental plaque; **D** is dilution fold of detection limit; **E**_**r**_ is relative PCR amplification efficiency.

An additional issue is that the color density may depend on the amount of DNA molecules, as well as other factors, including the amount of latex microspheres and oligonucleotides impregnated on the test lines; however, once a system is standardized, quantification could still be achieved by rigorously controlling the whole procedure.

**Figure 5:**
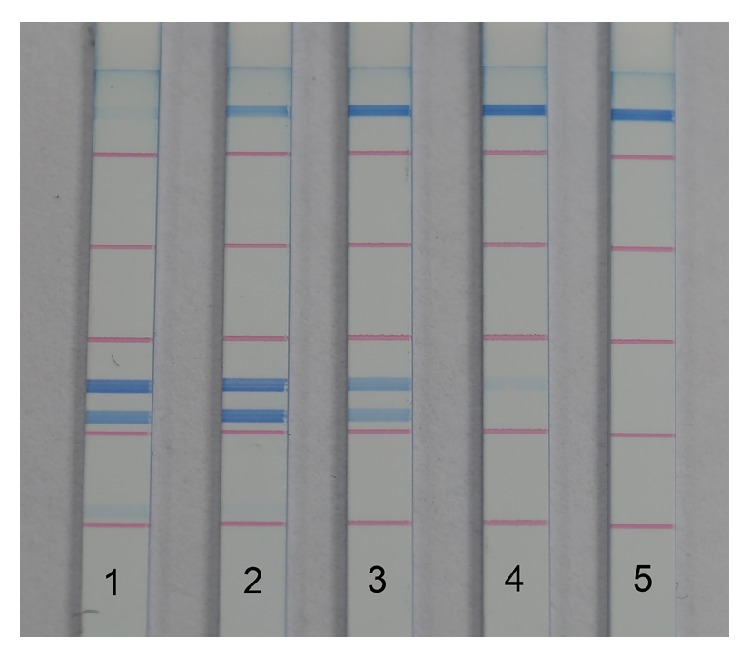
Semiquantitative chromatogram of sample 1. 1 *μ*L-aliquot for each specific PCR amplification was detected. Dilution factors for strips 1–5: original concentration, 10-fold, 10^2^-fold, 10^3^-fold, and 10^4^-fold, respectively.

PCR-dipstick DNA chromatography assay was applied to 16 supragingival plaque samples. A chromatogram (Figure 5) was obtained for each sample.* Actinomyces* species was detected in all 16 samples with unanimously high DNA concentrations.* S. mutans* and* V. parvula* presented in 15 of 16 samples. While frequently detected, these two species varied significantly in amount among samples.* S. sobrinus* was detected in only 1 sample with the lowest detection frequency, followed by* S. wiggsiae* with 4 positive samples out of 16. The positive species for each sample were enumerated. At least 2 of the 5 species tested were detected in the supragingival plaque samples.

Semiquantitative analysis was carried out based on the above calculation (Table 4). From relative concentration of each bacterial strain, the composition of the oral microbiota could be investigated simply and rapidly. Taking sample 1 as an example (Figure 5),* S. sobrinus* and* S. wiggsiae* were absent. Among the 3 positively detected species,* V. parvula* predominated over* Actinomyces* species and* S. mutans*. The relative concentrations of* S. mutans*,* Actinomyces* species, and* V. parvula* were estimated to be 1 : 10^5^ : 10^5^.* Actinomyces* species and* V. parvula* were observed to be the predominant bacteria in all tested samples.* S. mutans* exhibited higher detection frequency, but lower proportion among the whole plaque bacteria when compared with previous reports [12, 47], which could be attributed to the low detection limit of PCR-dipstick DNA chromatography and the use of different primers. There were no significant differences among subjects with low, moderate, and high DMFT (Table 4) in the detection frequencies (and amounts) of the five bacteria.

## 4. Conclusions

A disposable, paper-based PCR-dipstick DNA chromatography method was developed for simple, rapid and multiplex analysis of bacterial profiles in microbiota by impregnating several oligonucleotides on one strip to specifically recognize target DNA sequences. Semiquantitative information on the amount of target bacterial DNA in dental plaque can be obtained from dilution fold for detection limit of each bacterial species with correction using relative PCR efficiency of each bacterial species. The turnaround time from sampling to semiquantification requires only 3 h, and this time could be further compressed by refining the PCR method. This method can be applied to analysis of microbiota, such as in the oral cavity and intestinal tract, and to point-of-care diagnosis of microbiota-associated diseases such as dental caries and periodontitis. This universally designed method may be modified for a variety of purposes.

## Figures and Tables

**Table 1:** PCR primers and conditions.

Bacterial strain	Targeted gene	Primer name	Sequence (5′-3′)	Size (bp)	Annealing temperature (°C)
*S. mutans *	16S rRNA genes	Sm 1 (575F)	Tag 1-X-GGTCAGGAAAGTCTGGAGTAAAAGGCTA	282	56
		Sm 2 (856R)	Biotin-GCGGTAGCTCCGGCACTAAGCC		

*S. sobrinus *	16S rRNA genes	SobF (54F)	Tag 2-X-CGGACTTGCTCCAGTGTTACTAA	546	56
		SobR (599R)	Biotin-GCCTTTAACTTCAGACTTAC		

*S. wiggsiae *	16S rRNA genes	Scar 448F	Tag 3-X-GTGGACTTTATCAATAAGC	172	51
		Scar 619R	Biotin-CTACCGTTAAGCAGTAAG		

*Actinomyces *species	16S rRNA genes	AorF	Tag 4-X-GGCTGCGATACCGTGAGG	104	56
		AorR	Biotin-TCTGCGATTACTAGCGACTCC		

*V. parvula *	rpoB genes	Vp2696F	Tag 5-X-GAAGCATTGGAAGCGAAAGTTTCG	623	57
		Vp3318R	Biotin-GTGTAACAAGGGAGTACGGACC		

*Actinomyces* species includes *A. oris*, *A. naeslundii*, and *A. odontolyticus*.

“X” represents “spacer C3.” The Spacer C3 [three-carbon spacer, (CH_2_)_3_, using Phosphoramide C3 spacer (Glen Research, Sterling, VA)] is inserted between Tags 1 (to 5) and a respective forward primer [35].

**Table 2:** Procedure for semiquantification of plaque samples.

Procedure	Item	Amount (volume)
Plaque sampling	Dental plaque	1.5 mg

DNA extraction	InstaGene Matrix Kit	300 *µ*L

PCR	DNA extraction used for PCR	21 *µ*L
	Primer F (tagged) (5 *µ*M)	2 *µ*L
	Primer R (biotinylated) (5 *µ*M)	2 *µ*L
	Hot StarTaq PLUS Master Mix	25 *µ*L

Dipstick DNA chromatography	DNA amplified by PCR	1 *µ*L
	Deionized water	29 *µ*L
	Eluent **(supplied by TBA Co.; containing detergents, blocking agents, PBS, and salts solution)**	10 *µ*L
	Streptavidin-coated blue latex suspension **(supplied by TBA Co.)**	2 *µ*L

**Table 3:** Detection limit of PCR-dipstick DNA chromatography and PCR-agarose gel electrophoresis.

		100 pg	10 pg	1 pg	100 fg	10 fg	1 fg
*S. mutans *	Dip	+	+	+	+	+	+
	Aga	+	+	+	−	−	−

*S. sobrinus *	Dip	+	+	−	−	−	−
	Aga	+	−	−	−	−	−

*S. wiggsiae *	Dip	+	+	+	−	−	−
	Aga	+	+	−	−	−	−

*Actinomyces *species	Dip	+	+	+	−	−	−
	Aga	+	+	−	−	−	−

*V. parvula *	Dip	+	+	+	+	−	−
	Aga	+	+	+	−	−	−

**Table 4:** Semiquantification of five given bacteria in 16 supragingival plaque samples.

	Subject									Subject				Subject						
	1	2	3	4	5	6	7	8	Number of positive samples (%)	9	10	11	Number of positive samples (%)	12	13	14	15	16	Number of positive samples (%)	Number of positive samples for all subjects (%)
	Low DMFT								Moderate DMFT			High DMFT				
	0	0	0	0	0	1	2	2		3	4	4		8	10	12	12	12		
*S. mutans *	—	10^2^	1	10	10^3^	10	10	10^2^	7 (87.5)	10	10	1	3 (100)	1	10	10^2^	10^2^	10	5 (100)	15 (93.8)
*S. sobrinus *	—	—	—	—	—	—	—	—	0 (0.0)	—	—	—	0 (0.0)	—	—	10^5^	—	—	1 (20.0)	1 (6.3)
*S. wiggsiae *	—	—	—	—	10^5^	—	—	10^3^	2 (25.0)	—	—	—	0 (0.0)	—	—	10^3^	10^4^	—	2 (40.0)	4 (25.0)
*Actinomyces *species	10^5^	10^6^	10^5^	10^6^	10^6^	10^6^	10^5^	10^5^	8 (100)	10^6^	10^6^	10^5^	3 (100)	10^5^	10^6^	10^5^	10^5^	10^6^	5 (100)	16 (100)
*V. parvula *	10^4^	10^5^	10^4^	10^4^	10^5^	10^5^	10^3^	10^4^	8 (100)	—	10^2^	10^2^	2 (66.7)	10^5^	10^2^	10^4^	10^4^	10^2^	5 (100)	15 (93.8)

Number of positive bacterial species	2	3	3	3	4	3	3	4		2	3	3		3	3	5	4	3		
